# Preoperative predictors of unplanned conversion in laparoscopic liver resection: a multicenter cohort analysis

**DOI:** 10.3389/fonc.2025.1678775

**Published:** 2025-12-19

**Authors:** Yin Jiang, Dipesh Kumar Yadav, Gaoqing Wang, Zhekang Jiang, Shiwei Zhang, Gong Cheng, Xinhua Zhou, Haibiao Wang, Hong Li, Yiren Hu, Yongfei Hua

**Affiliations:** 1 Department of Hepatobiliary and Pancreatic Surgery, Ningbo Medical Center Lihuili Hospital, Ningbo University, Ningbo, Zhejiang, China; 2 Department of General Surgery, Wenzhou People’s Hospital, The Third Clinical Institute Affiliated to Wenzhou Medical University, Wenzhou, Zhejiang, China; 3 Department of Health Science Center, Ningbo University, Ningbo, Zhejiang, China

**Keywords:** conversion, laparoscopy, liver resection, liver tumor, risk factors

## Abstract

**Objective:**

To identify preoperative predictors of conversion from laparoscopic to open hepatectomy for optimized patient selection.

**Methods:**

This retrospective cohort study analyzed 755 patients undergoing laparoscopic liver tumor resection at two tertiary centers (December 2019–June 2025). Patients were stratified by surgical approach: laparoscopic completion (n=709) versus unplanned conversion (n=46). Univariate analysis was performed using the chi-square (χ²) test for categorical variables and the independent samples t-test for continuous variables. Variables with a p-value < 0.05 were included in multivariate logistic regression analysis to identify independent risk factors for conversion. A p-value of <0.05 was considered statistically significant.

**Results:**

The conversion rate was 6.09% (46/755), predominantly due to uncontrolled bleeding (43.5%) and severe adhesions (34.8%). Multivariate analysis identified five independent predictors: history of abdominal surgery (OR = 2.12, 95%CI: 1.05–4.26); liver cirrhosis (OR = 5.34, 95%CI: 1.80–15.84); postero-superior tumor location (OR = 11.36, 95%CI: 5.49–23.52); extended resection (≥3 segments, OR = 2.80, 95%CI: 1.22–6.43); and extrahepatic organ resection (OR = 4.71, 95%CI: 1.13–19.56). Notably, while larger tumor showed univariate significance (p=0.041), it was not an independent multivariate predictor.

**Conclusion:**

Critical preoperative risk factors for conversion include history of abdominal surgery, liver cirrhosis, tumors located in the postero-superior segment, extensive liver resection, and liver resection combined with extrahepatic organ resection. Careful selection of appropriate candidates for laparoscopic liver resection can help reduce the risk of conversion to laparotomy and the occurrence of further complications.

## Introduction

In hepatic surgery, the seminal work by Reich et al. (1991) marked the inception of laparoscopic resection for benign hepatic neoplasms (1). Despite its potential, integration into clinical practice progressed gradually, constrained by procedural technical complexity and concerns regarding perioperative risk management, particularly for malignant tumors (2, 3). However, recent advancements in surgical techniques and technology have substantially enhanced the acceptance and credibility of laparoscopic liver resection within the surgical community. Compared to open hepatectomy, laparoscopic resection offers significant advantages, including reduced tissue trauma, diminished intraoperative blood loss, lower postoperative morbidity, and shorter hospital stays. Furthermore, its therapeutic efficacy has proven comparable to open surgery, solidifying its status as a viable alternative (4).

The indications for laparoscopic hepatectomy continue to broaden (5–9). Nevertheless, unplanned conversion to open surgery remains a possibility, with reported rates ranging from 1.74% to 16.6%, primarily due to significant intraoperative haemorrhage (10–14). Conversion not only prolongs operative duration but also increases surgical risks. This critical decision is influenced by the surgeon’s laparoscopic expertise and various preoperative factors (15).

While prior research has addressed this topic, the existing literature warrants further exploration. This study aims to enhance current understanding by conducting a comprehensive analysis of preoperative risk factors for conversion, utilizing a large patient cohort to provide actionable insights. By elucidating these factors, we seek to mitigate surgical risks and optimize patient selection for laparoscopic hepatectomy.

## Patients and methods

### Patients

A retrospective review included patients undergoing laparoscopic liver resection at Ningbo Medical Center LiHuiLi Hospital and Wenzhou People’s Hospital between December 2019 and June 2025. The study protocol received approval from the institutional ethics committees of both centers and adhered to the ethical standards of the Declaration of Helsinki (16). Written informed consent was obtained preoperatively from all patients or their legal representatives.

The cohort comprised 755 patients (469 male, 286 female; mean age 58.88 years). Exclusion criteria encompassed: (1) preoperatively planned conversion to laparotomy, (2) history of liver transplantation, and (3) severe comorbidities (cardiac, pulmonary, renal, or coagulation disorders). Eligible patients were dichotomized by final surgical approach: the laparoscopic group (n=709) and the conversion group (n=46).

Data were extracted from the Electronic Medical Record System (EMRS) using a standardized Excel template. The collected variables encompassed patient demographics, body mass index (BMI), comorbid conditions such as hypertension and diabetes, the American Society of Anesthesiologists (ASA) physical status classification, history of abdominal surgery, status of hepatitis B, presence of liver cirrhosis, Child-Pugh score, and post-operative pathology reports that classified the tumors. In addition, the maximum tumor diameter, number of tumors, tumor location, and the intended resection margins were documented.

### Per-operative evaluation of patients for surgery

All patients underwent comprehensive preoperative assessment with contrast-enhanced abdominal computed tomography (CECT) and magnetic resonance imaging (MRI). These imaging techniques were essential to delineate tumor characteristics, including precise location, dimensions, type, and spatial relationships with adjacent structures (e.g., vasculature, biliary ducts, and surrounding organs). Challenging cases were reviewed in multidisciplinary team (MDT) conferences prior to surgery.

### Surgical technique

#### Laparoscopic hepatectomy

Following induction of general anesthesia, patients were positioned supine with 15°–45° reverse Trendelenburg tilt and right-side elevation to optimize hepatic exposure. A 10-mm trocar was inserted via an infraumbilical incision to establish pneumoperitoneum (13–15 mmHg), followed by introduction of a 30° laparoscope for systematic abdominal inspection to exclude incidental pathology or iatrogenic injury. Four additional ports were placed under laparoscopic guidance: a subxiphoid port for liver retraction, 12-mm working ports bilaterally at midclavicular lines (umbilical level), and two 5-mm accessory ports (midline supraumbilical and right anterior axillary line), ensuring optimal instrument triangulation and ergonomics. Liver mobilization involved division of the falciform, coronary, and triangular ligaments to expose the target segment. Resection strategy (anatomic vs. non-anatomic) was determined by tumor location: anatomic resection included ligation of segmental hepatic veins and portal pedicles, while non-anatomic resection entailed precise tumor excision with parenchymal margins.

Parenchymal transection was performed using an ultrasonic harmonic scalpel, with routine application of the Pringle maneuver for inflow control. Vascular and biliary structures were secured with clips or polypropylene sutures. Intraoperative ultrasound provided real-time guidance to verify adequate resection margins and confirm complete tumor clearance during parenchymal transection. Resected specimens were retrieved in sterile bags via Pfannenstiel incision. Procedures concluded with peritoneal irrigation and placement of passive drains through port sites.

#### Conversion to laparotomy

When conversion was required, open resection employed conventional techniques (17). The Pringle maneuver controlled hemorrhage during parenchymal transection. Dissection utilized an ultrasonic harmonic scalpel for coagulation/cutting, Cavitron Ultrasonic Surgical Aspirator (CUSA) for precise parenchymal dissection, Endo-GIA™ staplers for hepatic vein division, and argon beam coagulator for surface hemostasis.

### Postoperative care

Postoperative care included vigilant monitoring, pain management, respiratory function assessment, and early mobilization to promote recovery and reduce complications. Patients were monitored for hemorrhage, bile leak, or infection, with prompt intervention for deviations from the expected postoperative course.

### Definitions

Liver segments were classified according to the Couinaud system: the anterolateral sector comprised segments II, III, IVb, V, and VI, while the postero-superior sector included segments I, IVa, VII, and VIII. Resection extent followed Brisbane 2000 terminology, with “extended liver resection” defined as resection of three or more segments (18). Conversion to laparotomy was defined as any unplanned transition to open surgery during the laparoscopic procedure. Previous upper abdominal surgery referred to any prior open or laparoscopic procedure involving abdominal organs. Maximum tumor diameter was defined as the longest unidimensional measurement (cm) on preoperative contrast-enhanced CT/MRI across axial, coronal, or sagittal planes. Tumor staging adhered to the 8th edition AJCC/UICC TNM classification. Extrahepatic organ resection was defined as resection of any abdominal organ except the gallbladder.

### Statistical analysis

Data were analyzed using SPSS 22.0 (IBM Corp., Armonk, NY). Continuous variables are presented as mean ± standard deviation (SD), and categorical variables as absolute frequencies (n). Univariate analysis employed χ² tests for categorical variables and independent t-tests for continuous variables. Variables with p < 0.05 in univariate analysis were entered into a multivariate logistic regression model using backward elimination to identify independent predictors of conversion. Statistical significance was defined as p < 0.05.

## Results

Postoperative pathological outcomes for the 755 patients undergoing liver tumor resection are detailed in **Table 1**. The overall conversion rate to laparotomy was 6.09% (46/755). Primary indications for conversion included uncontrolled intraoperative bleeding (n=20, 43.5%), severe adhesions (n=16, 34.8%), exposure limitations (n=8, 17.4%), and intraoperative rupture of malignant tumors (n=2, 4.3%).

**Table 1:** Postoperative pathological results.

Indication for resection	N
Primary hepatocellular carcinoma	406
Hepatic hemangioma	146
Other benign liver tumors (bile duct cystadenoma, hepatic adenoma, angiomyolipoma, etc.)	75
Intrahepatic cholangiocarcinoma	43
Colorectal cancer liver metastasis	39
Liver metastasis from other tumors	39
Mixed liver cancer	6
Liver sarcoma	1

Univariate analysis demonstrated no statistically significant differences between the laparoscopic (n=709) and conversion (n=46) groups in age, BMI, comorbidities (hypertension and/or diabetes), ASA grade, hepatitis B status, Child-Pugh score, and number of tumors (all P > 0.05). Significant differences emerged for: history of abdominal surgery (χ²=4.44, P = 0.035), liver cirrhosis (χ²=8.52, P = 0.004), malignant pathology (χ²=6.46, P = 0.04), larger tumor (t=2.0, P = 0.041), tumor located in the postero-superior segment (χ² = 69.84, P < 0.001), extensive liver resection (χ² = 11.34, P = 0.001), and liver resection combined with extrahepatic organ resection (χ² = 4.44, P = 0.035) (**Table 2**).

**Table 2:** Comparative analysis of clinical characteristics in patients undergoing laparoscopic liver tumor resection- conversion vs. non-conversion groups.

Variable	Laparoscopy group (n = 709)	Conversion group (n = 46)	x2 value/t-value	P value
Age (years)			0.21	0.648
≥70	143 (20.2)	8 (17.4)		
<70	506 (79.8)	38 (82.6)		
BMI (kg/m²)			1.94	0.164
≥28	54 (7.7)	1 (2. 2)		
<28	647 (92.3)	45 (97. 8)		
Comorbidities (hypertension and/or diabetes)			0.001	0.978
Yes	353 (49.8)	23 (50)		
No	356 (50.2)	23 (50)		
ASA grade			1.87	0.393
Class 1	127 (17.9)	5 (10.9)		
Class 2	577 (81.4)	41 (89.1)		
Class 3	5 (0.7)	0 (0)		
History of abdominal surgery			4.44	0.035
Yes	218 (30.7)	21 (45.7)		
No	491 (69.3)	25 (54.3)		
Hepatitis B			0.03	0.856
Yes	391 (55.1)	26 (56.5)		
No	318 (44.9)	20 (43.5)		
Liver cirrhosis			8.52	0.004
Yes	446 (62.9)	19 (41.3)		
No	263 (37.1)	27 (58.7)		
Child-Pugh classification			0.13	0.718
Child A	707 (99.7)	46 (100)		
Child B	2 (0.3)	0 (0)		
Pathological results			6.46	0.04
Primary malignant tumor	427 (60.2)	29 (63)		
Benign tumor	213 (30.0)	8 (17.4)		
Metastatic tumor	69 (9.7)	9 (19.6)		
Maximum tumor diameter (cm)	3.7 ± 2.1	4.4 ± 2.4	2.0	0.041
Number of tumors			0.67	0.415
Single	602 (84.9)	37 (80.4)		
≥2	107 (15.1)	9 (19.6)		
Tumor located in postero-superior segmenta			69.84	<0.001
Yes	151 (21.3)	35 (76.1)		
No	558 (78.7)	11 (23.9)		
Extensive liver resectionb			11.34	0.001
Yes	100 (14.2)	15 (32.6)		
No	606 (85.8)	31 (67.4)		
Liver resection combined with extrahepatic organ resection			4.44	0.035
Yes	21 (3)	4 (8.7)		
No	688 (97)	42 (91.3)		

a Posterior–superior segment is I, IVa, VII, VIII; b Extensive liver resection range of ≥3 liver segments; BMI, Body mass index; ASA, American society of anesthesiologists.

Multivariate logistic regression incorporating these significant variables identified five independent predictors of conversion: history of abdominal surgery (OR = 2.12, 95% CI: 1.05–4.26), liver cirrhosis (OR = 5.34, 95% CI: 1.80–15.84), tumor location in the postero-superior segment (OR = 11.36, 95% CI: 5.49–23.52), extensive liver resection (OR = 2.8, 95% CI: 1.22–6.43), and liver resection combined with extrahepatic organ resection (OR = 4.71, 95% CI: 1.13–19.56) (all P < 0.05, **Table 3**).

**Table 3:** Multivariate analysis (Backward elimination method) of risk factors for conversion to open hepatectomy in patients undergoing laparoscopic liver tumor resection.

Variable	β	S.E.	Wald χ2	OR	95% CI	P value
History of abdominal surgery	0.82	0.37	5.06	2.12	1.05~4.26	0.036
Liver cirrhosis	1.67	0.56	9.10	5.34	1.80~15.84	0.003
Tumor located in postero-superior segmenta	2.43	0.37	42.92	11.36	5.49~23.52	<0.001
Extensive liver resection	1.03	0.42	5.93	2.8	1.22~6.43	0.015
Liver resection combined with extrahepatic organ resection	1.55	0.73	4.55	4.71	1.13-19.56	0.033

β, Regression coefficient; S.E., Standard error of regression coefficient; CI, Confidence interval; OR, Odds ratio.

## Discussion

Laparoscopic liver resection is widely established as the standard surgical approach for tumors in the left lateral liver segment (6). Over the past two decades, laparoscopic techniques have rapidly expanded to encompass more complex procedures including caudate lobe resection (19), two-step hepatectomy with portal vein ligation (20), and living donor liver transplantation (7). Despite these technical advancements, conversion rates remain clinically significant, ranging from 7% to 16.6% in contemporary literature (10–13). Our study observed a conversion rate of 6.09% (46/755), aligning with these reported figures. Notably, Jo et al. documented an exceptionally low conversion rate of 1.74% among 1,951 patients (14), suggesting potential for further refinement of surgical approaches.

Previous investigations have consistently identified tumor located in the postero-superior segment, advanced age (≥75 years), diabetes mellitus, hypertension, elevated BMI (≥28 kg/m²), tumor diameter exceeding 10 cm, biliary remodeling, and major liver resections (including right hepatectomy) as risk factors for conversion (10–12, 21). Our analysis confirmed tumor located in the postero-superior segment and extensive liver resection as independent risk factors while contributing novel evidence that history of abdominal surgery, liver cirrhosis, and liver resection combined with extrahepatic organ resection independently predict conversion. Interestingly, despite larger tumor demonstrating significance in univariate analysis (P = 0.041), it did not emerge as an independent predictor in multivariate modeling. This finding suggests that the apparent association between tumor size and conversion may be mediated through its relationship with other variables such as tumor location or resection complexity rather than representing a direct causal factor. Additionally, contrary to some expectations, advanced age, comorbidities (hypertension/diabetes), and a BMI ≥28 kg/m² showed no significant association with conversion risk in our cohort.

The magnitude of risk elevation associated with these identified independent factors warrants emphasis: patients with a history of abdominal surgery demonstrated a 2.12-fold higher risk of unplanned conversion, which was frequently necessitated by severe adhesions. Furthermore, the presence of liver cirrhosis conferred a 5.34-fold increased risk, while tumors located in the postero-superior segment escalated the risk by a substantial factor of 11.36, with intraoperative hemorrhage being the predominant challenge. Similarly, extensive liver resection was associated with a 2.8-fold increased risk, and liver resection combined with extrahepatic organ resection was linked to a 4.71-fold increased risk of conversion, as these complex procedures often presented difficulties with exposure and hemostasis.

The anatomical complexities of the postero-superior liver segment, which is in proximity to the second hepatic hilum, demand nuanced surgical manipulation. Scoring systems designed to evaluate the technical complexity of laparoscopic hepatectomies consistently assign higher difficulty scores to procedures involving this segment, reflecting its challenging anatomy (22–24). These challenges are compounded by the limited operating space and the constraints imposed by the costal margin, resulting in suboptimal exposure of deep hepatic structures and major hepatic vein tributaries. The complexity is further increased when tumors are located deeply, affecting the accuracy of visual tumor margin assessment and necessitating multi-planar dissection. Consequently, this anatomical region is predisposed to substantial hemorrhage that can be refractory to conventional control methods, thereby heightening the risk of intraoperative conversion to laparotomy.

To address these technical constraints, comprehensive preoperative vascular mapping using CT/MRI with 3D reconstruction is indispensable. Intraoperative strategies such as flexible laparoscopy, real-time ultrasound navigation, and indocyanine green (ICG) fluoroscopy significantly enhance margin visualization and vascular delineation. Innovative approaches like the parenchymal-sparing “Diamond technique” for deep postero-superior tumors (25) and anatomical resection methods (e.g., Glissonean-first and cranio-caudal approaches along Laennec’s capsule planes) (26–29) have demonstrated efficacy. Supplemental technical modifications including semi-prone positioning (30), intercostal port placement (31, 32), and transthoracic access (32) further facilitate exposure.

A history of abdominal surgery—particularly open procedures causing dense adhesions around the hepatoduodenal ligament—remains a significant conversion risk. Cipriani et al. reported substantially higher conversion rates in these patients (13.7% vs 5.1%, P = 0.021) (33), though advanced laparoscopic techniques now enable successful resection in selected cases (34). Meticulous adhesiolysis is paramount to prevent vascular or biliary injury during dissection.

Compared with small-scale liver resection, large-scale liver resection combined with resection of multiple organs is associated with increased procedural complexity, larger wound surfaces, prolonged operative durations, and an elevated risk of hemorrhage. Similarly, patients with liver cirrhosis may be particularly ill-equipped to withstand the physiological stress of extensive liver resections due to their diminished liver reserve and enhanced propensity for bleeding (35). Although numerous centers have validated the safety of laparoscopic liver resection in cirrhotic patients (36, 37), nevertheless, a thorough and cautious preoperative assessment remains a prerequisite for patient selection in anticipation of laparoscopic liver resection.

Consistent with prior studies (10–12), uncontrolled intra-operative bleeding was the most common cause of conversion in our study, accounting for 43.4% (20/40) of cases. Apart from the surgeons’ learning curve in laparoscopic liver resection, the leading causes of intra-operative bleeding in our study were as discussed earlier, i.e., postero-superior segment resection, severe adhesion due to previous surgery, extensive liver resection, combined resection of multiple organs, and liver cirrhosis. Optimal visibility of the surgical field, adequate preparation of hemostasis instruments, and prompt control of bleeding are essential for a safe and successful laparoscopic liver resection and can help reduce the risk of unplanned conversion.

The literature describes several well-established techniques to minimize and control bleeding during laparoscopic liver resection. These include inflow control via the Pringle maneuver and hemihepatic clamping or selective clamping of the portal vein. Similarly, outflow control can be achieved through the reverse Trendelenburg position—which reduces venous return from the lower extremities—and lowering central venous pressure (CVP) to ≤ 5 mmHg, which decreases venous engorgement of the liver and reduces backflow during parenchymal transection (38, 39). Additionally, increasing pneumoperitoneum pressure to 13–15 mmHg can also reduce bleeding from the liver transection area by exerting positive pressure on the portal venous system (38). However, these techniques must be used with caution, as CVP ≤ 5 mmHg increases the risk of gas embolism (40), while the reverse Trendelenburg position and elevated pneumoperitoneum pressure may cause cerebral infarction due to gas embolism and have been shown to negatively impact liver regeneration and renal perfusion (41–43).

Additionally, bleeding can be further controlled through several methods: compressing bleeding sites with a small gauze piece, applying endo-clips or sutures to bleeding vessels, and performing dorsal compression of liver parenchyma in cases of bleeding from the branches of hepatic veins. Techniques such as the modified hanging maneuver (44), anterior approach without liver mobilization (13), and hand-assisted laparoscopy or hybrid technique (45) have also been shown to be effective in controlling bleeding and increasing the safety of laparoscopic liver resection. In addition to the aforementioned techniques, intra-operative bleeding can also be effectively managed using a variety of surgical instruments. These include the harmonic scalpel, Endo-GIA stapler, Aquamantys system, argon-beam coagulator, water jet scalpel, cooled-tip radiofrequency probe (Habib’s Technique), bipolar cautery device, TissueLink monopolar floating ball (TMFB), and Chang’s needle (46). Importantly, it is imperative that the decision to convert to open surgery is made expeditiously in patients at high risk to avert exacerbation of blood loss and its associated complications.

Our study, while providing valuable insights, is not without potential limitations. Its retrospective nature predisposes it to selection bias. Additionally, our analysis did not encompass data pertaining to patients who received neoadjuvant therapy for liver cancer. We speculate that this exclusion likely influenced our results, potentially leading to an underestimation of the overall conversion rate. This is because patients requiring neoadjuvant therapy often present with more advanced disease, and the therapy itself induces microvascular changes (such as peliosis hepatis and sinusoidal congestion) (47). These factors collectively increase the risk of intraoperative hemorrhage and technically challenging dissections, which are key drivers for conversion to laparotomy. By omitting this higher-risk subgroup, our cohort may reflect a more favorable surgical population. Lastly, inter-surgeon variability in surgical techniques within our institutions may have confounded the study outcomes, and the surgeons’ learning curve cannot be ruled out. Despite these limitations, our investigation is pertinent and timely, offering a comprehensive analysis of a large patient cohort from a single center and meticulously examining clinically significant variables that may influence the decision to convert to laparotomy.

## Conclusions

In conclusion, despite advancements in surgical techniques, conversion to laparotomy during laparoscopic hepatectomy is an event that cannot be entirely prevented at present, meticulous patient selection based on the surgeon’s experience and recognized conversion risk factors is pivotal. Such a strategic approach can significantly mitigate the risk of conversion, thereby potentially reducing associated postoperative complications.

## Data Availability

The original contributions presented in the study are included in the article/supplementary material. Further inquiries can be directed to the corresponding authors.
